# Beyond the Dopaminergic System: Lessons Learned from levodopa Resistant Symptoms in Parkinson's Disease

**DOI:** 10.1002/mdc3.13786

**Published:** 2023-06-01

**Authors:** Angelo Antonini, Aron Emmi, Marta Campagnolo

**Affiliations:** ^1^ Parkinson and Movement Disorders Unit, Centre for Rare Neurological Diseases (ERN‐RND), Department of Neuroscience University of Padova Padova Italy; ^2^ Center for Neurodegenerative Diseases (CESNE) University of Padova Padova Italy; ^3^ Institute of Human Anatomy, Department of Neuroscience University of Padova Padova Italy

**Keywords:** l‐dopa, Parkinson, motor complications, progression

## Introduction

Diagnosis and clinical burden in people with Parkinson's disease (PD) is often related to the cardinal motor features (tremor, bradykinesia, rigidity) and occurs when at least 40–60% of striatal dopamine nerve terminals are lost.^1^ However, in the “Essay on the shaking palsy” James Parkinson already reported presence of constipation, cognitive dysfunction or sleep disorders in addition to motor involvement.^2^ With the introduction of l‐dopa therapy it became evident that only specific clinical features would improve while many others would show limited or no benefit from medications even if they significantly contribute to quality of life decline and disability. In recent years the detection of pathological hallmarks in many tissues beyond the central nervous system has supported the concept that PD is a systemic multi‐organ condition where dopamine is less centric and various neurotransmitters and pathways are involved.

In the prodromal phase (the time between onset of neurodegeneration and occurrence of motor symptoms), orthostatic hypotension (OH), constipation, hyposmia, rapid‐eye movement (REM) sleep behavior disorder (RBD), pain and neuropsychiatric symptoms (ie, subtle frontal lobe deficits and depression) may be observed.^3^ Severe autonomic dysfunction and cognitive impairment significantly contribute to clinical deterioration in the advanced disease stages.^4^

**FIG. 1:**
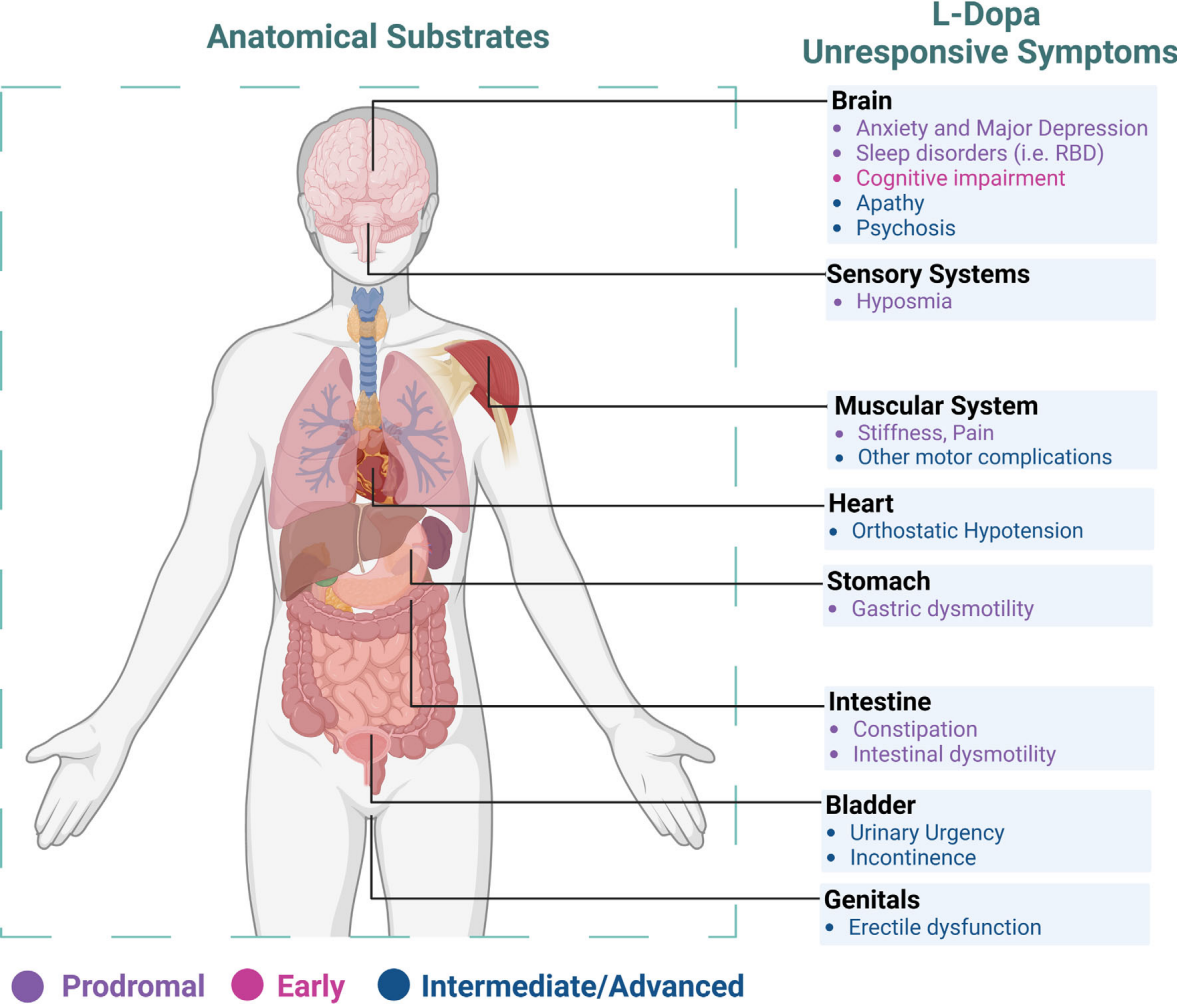
Shows anatomical regions and related l‐dopa non‐responsive or partially‐responsive symptoms. The colors refer to the disease stage.

In this Viewpoint, we aimed to challenge the traditional vision of PD motor and non‐motor dichotomy. We have reviewed the most relevant PD features and the respective neurotransmitters’ systems involved, suggesting a categorization based on response to dopamine replacement therapy. We believe these considerations are relevant in light of the possibility to detect and possibly treat the disease years before motor manifestations (Fig. 1).

## l‐dopa Responsive Symptoms

### Prodromal Phase

#### Oculo‐Visual Dysfunction

Innervation in the retina (especially around the fovea) is mainly dopaminergic, and therefore vision dysfunction is common in PD. It is often expressed as difficulties in color and contrast vision during off periods which may benefit from treatment optimization.^5^ Diplopia is often not corrected by dopaminergic treatment and it may develop from a combination of convergence insufficiency and exophoria.

#### Pain

Shoulder pain is frequently reported at onset of motor manifestations but this is generally secondary to limb rigidity and progressively improves once dopaminergic therapy is established.^5^

#### Cardinal Motor Features

Bradykinesia and rigidity usually present a satisfying and sustained response to l‐dopa while rest‐tremor may be more difficult to treat in some patient.^6^

### Advanced Phase

#### Motor Manifestations and Complications

Medications‐related fluctuations, wearing off and dyskinesias develop after a few years of therapy in patients with good response to l‐dopa. Variability in brain dopamine levels secondary to intermittent l‐dopa administration, heterogeneous gastrointestinal function and absorption, progressive loss of dopamine nerve terminals and storage capacity contribute to the development of these symptoms.^7^, ^8^ Therapeutic strategies aiming at more constant drug delivery and dopamine receptor stimulation are helpful and if started early, may minimize the severity of motor complications.^7^

#### Urinary Urgency and Incontinence

The dopaminergic pathway plays a key role in the regulation of the genitourinary system. Dopamine selectively inhibits or activates the pontine micturition center, explaining the frequent urinary symptoms in PD. Optimizing dopaminergic treatment may help in the management of the urinary urgency secondary to detrusor overactivity in PD.^5^ However, early occurrence of urinary urgency and bladder dysfunction may also express more rapid disease progression and deserves careful assessment by treating physicians.

## l‐dopa Non‐Responsive Symptoms

l‐dopa non‐responsive symptoms are common and may be observed already at prodromal phase. They are generally related to either misfolded alpha‐synuclein accumulation in various tissues including the dopamine pathways (structural alterations) or consequence of dysfunctions affecting other neurotransmitters (functional alterations).

### Prodromal Phase

#### Hyposmia

Olfactory dysfunction occurs months or years before motor onset and it is associated with fourfold increase in risk of PD within 4–10 years.^9^ However, it is often overlooked by clinicians and patients, and sometimes is confused with the physiological age‐related olfactory decline.

The lack of response to l‐dopa supports the concept that hyposmia is not related to the dopamine system and generally does not follow a parallel progression with motor decline.^10^ Abnormalities have been observed in the central olfactory structures, whereas the involvement of the peripheral olfactory system is less defined. Lewy bodies are commonly found in these regions, supporting the hypothesis of pathology starting in the periphery then extending to the brain.^10^, ^11^ Since the olfactory system directly links the brain to the external environment, it could be a gateway through which many viruses, including novel coronavirus, could trigger inflammation and ultimately promote neurodegenerative processes.^12^

#### Constipation

Constipation is another common PD risk factor. There is increasing interest regarding the role of the gut–brain axis, and both alpha‐synuclein aggregates and reactive enteric gliosis have been detected in nerve fibers of the duodenal mucosa, in both early and advanced PD.^13^, ^14^, ^15^ In addition, presence of constipation is associated with faster progression and involves the cholinergic system.^16^ Finally, alterations in the gut microbiota composition are also well documented supporting interventions modulating gut microbiota.^17^

#### Pain

Generalized painful sensations or dystonic pain may develop in patients with motor fluctuations, off periods and peak‐dose dyskinesias, as well as in association to restless legs syndrome or periodic limb movement. Given the key role of dopamine in mediating several pain networks in the central nervous system (spinal cord, thalamus, basal ganglia, cingulate cortex), advanced patients suffering from pain often benefit from an optimized dopaminergic therapy.^5^ However, many patients suffer from neuropathic and central pain independently from motor complications suggesting involvement of other neurotransmitters including glutamate, norepinephrine and serotonin. A broader approach to pain management that considers multiple drugs including opiate is warranted given its relevance to quality of life.^18^

### Early PD Phase

#### Cognitive Impairment

Mild cognitive deficits in executive functions, working memory, attention, difficulties in planning and problem solving are common early PD features. Dementia is reported in PD with long survival and its occurrence is underpinned by cholinergic dysfunction, diffuse Lewy body pathology and frequently associated with amyloid pathology in elderly patients.^19^ Rivastigmine, an acetylcholinesterase inhibitor, may improve executive function, attention and behavioral symptoms in PD patients with dementia suggesting a role for cholinergic drugs. Presence of different regional patterns of cholinergic dysfunction may explain various l‐dopa unresponsive features including cognitive decline.^20^

### Intermediate/Advanced PD Phase

#### Postural Instability or Abnormalities, Freezing, Falls

Gait difficulties and postural instability are amongst the most disabling PD features and often overlap with cognitive impairment resulting in rapid loss of autonomy and worsening quality of life. The unsatisfying response to dopaminergic treatment and device aided therapies suggests involvement of non‐dopaminergic networks and nuclei.^21^

#### Dyskinesias

Abnormal striatal plasticity and pharmacodynamic changes in dopaminergic transmission are the neurobiological substrates of dyskinesia. l‐dopa treatment determines internalization of A2A‐D2 heteromers and compensatory upregulation of A2A homomers, ultimately leading to an impaired firing pattern in the striato‐pallidal pathway.^22^ Hence, management of dyskinesia is challenging and requires a diversified treatment targeting different neurotransmitters especially glutamatergic modulation (ie, amantadine, a NMDA receptor antagonist) or implementation of device aided therapies. Strategies targeting serotonin have not been successful in patients although animal models suggest a role for this neurotransmitter in the development of dyskinesia.

#### Behavioral symptoms (psychosis, impulse control disorder [ICD])

Psychosis is characterized by hallucinations, delusions (false beliefs against contrary knowledge) and delirium. They occur in the context of cognitive decline and dementia but may be also triggered by dopaminergic therapy.^23^ Modulation of dopaminergic therapy along with use of atypical antipsychotic medications including pimavanserin (currently available only in the USA) should represent the treatment of choice, as older antipsychotic medications often led to worsening of motor manifestations.

Impulsivity and ICD are more common in patients on DA replacement treatment especially dopamine agonists.^24^, ^25^, ^26^ This supports the notion that ICD is related to dopaminergic dysfunction and can be managed only by modulation of dopamine replacement therapy.

#### Orthostatic Hypotension

OH has an estimated prevalence ranging from 30–65% of PD patients. It is reported occasionally as a prodromal feature but more frequently detected across all stages of the disease. The main role played by noradrenaline in modulating the autonomic function explains why the disruption of the noradrenergic pathways in PD underlines the cardiovascular abnormalities including OH. Extracardiac, and more importantly, cardiac sympathetic denervation are frequently associated with additional dysfunction in the parasympathetic compartment and impaired baroreflex.^27^ To date, a standardized approach to OH is lacking and the relationship between dopamine and OH is still debated,^28^ together with the inclusion of OH among potential side effects of levodopa and dopamine agonists, non‐pharmacological treatments are often recommended as the first choice.

## Manifestations Involving Both Dopaminergic and Non‐Dopaminergic Pathways

These manifestations result from the complex interplay between multiple neurotransmitter systems, and may partially benefit from dopaminergic treatment. Indeed, this category comprises manifestations presenting motor, cognitive and behavioral components, likely reflecting the involvement of multiple anatomical and neurochemical circuits.

### Prodromal Phase

#### REM Sleep Behavior Disorder

RBD is a strong risk factor and predictor of PD and other synucleinopathies (25–40% at 5 years, 40–65% at 10 years).^13^, ^29^ Several neuroimaging and functional studies support the role of dopaminergic dysfunction and nigrostriatal degeneration in the genesis of RBD although the involvement of other neurotransmitters is likely since RBD is often unresponsive to dopaminergic treatment alone.^30^ RBD generally occurs many years before motor manifestations and the role of noradrenergic depletion of the locus coeruleus has been documented by many studies.^31^ In support of this hypothesis, a recent study suggested the efficacy of safinamide, a medication with both dopaminergic and nondopaminergic actions (inhibition of monoamine oxidase‐B, modulation of glutamate release) in improving symptoms related to RBD.^32^

#### Anxiety and Major Depression

Depression or depression‐related symptoms are listed among the criteria for Prodromal PD.^33^ The presence of comorbid anxiety disorders is also common and may occur as a separate entity or in relation to motor fluctuations especially during the off periods. In addition to dopamine, extra‐striatal pathology and degeneration in the serotonergic and noradrenergic afferent pathways from the midbrain play an important role in PD depression.^34^ In addition, reactive behavioral patterns, symptoms occurring during motor worsening or as drug adverse effects, need to be considered when approaching treatment. There is no randomized clinical trials on the antidepressant effects of l‐dopa and one randomized placebo controlled study documenting the effect of pramipexole.^35^ However, in some PD patients depressive symptoms may not benefit from dopaminergic therapy and they require additional drugs targeting the serotonergic and/or noradrenergic system especially if anxiety or apathetic features are present.^5^

### Advanced Phase

#### Apathy

Apathy can be observed alone or in association with depression or anxiety. A prominent dopaminergic and noradrenergic dysregulation are relevant causative factors in determining apathy and should both be considered when introducing treatment. Apathy may also be a strong predictor of cognitive decline and its persistence should require careful monitoring of neuropsychological skills.^36^

## Conclusions

An increasing body of work supports the view of PD as a systemic condition with multiple organs and neurotransmitters involved and where symptoms poorly addressed by l‐dopa therapy have a key role on quality of life, autonomy and functional decline.^5^, ^37^ Moreover, l‐dopa resistant symptoms constitute a major target for advanced and palliative treatments in the late stage of disease.^38^ Given the traditional focus on dopamine, many of these manifestations are often overlooked although they significantly impact on the global progression of the disease, life expectancy and on the cost of care.^5^

Indeed patients presenting early multidomain cognitive impairment or RBD may suffer more rapid physical decline as opposed to individuals who manifest predominant mild motor and mood dysfunction where disease course is generally more benign. Similar prominent early autonomic deficits including OH and constipation have been associated with worse prognosis, higher risk of cognitive impairment, functional decline and death.^39^

Overall these findings support the redefinition of PD as a systemic neurodegenerative process involving different systems but with different rates of degeneration and progression. l‐dopa unresponsive symptoms present a heterogeneous progression pattern, that does not mirror classic and well‐documented motor decline. Identification and tracking of those symptoms should parallel motor evaluation to properly define prognosis.^37^, ^39^

The neuropathological hallmarks of many l‐dopa unresponsive features might be related to alpha synuclein misfolding and aggregation involving various neurotransmitters and functional networks in addition to nigrostriatal dopamine neurons.^11^ Indeed, the presence of overlapping pathological processes (amyloid and Tau) or concomitant cerebrovascular disease is increasingly relevant and documents the heterogeneity of the disease and the need of more patient centric therapeutic approaches.

Current “clinical markers” of disease are heavily weighted towards the nigrostriatal dopaminergic system but we believe the objective of pharmacological research should expand to other neurotransmitter systems, particularly on compounds targeting serotoninergic, glutamatergic and purinergic pathways.

Our suggestion is that a new approach to PD diagnosis and management beyond dopamine replacement therapy should be rapidly adopted, encompassing all clinical features and addressing their contribution to early disease detection, prognosis, progression and quality of life. Recent discoveries in disease biomarkers indicate that it is important to change our dopamine‐centric perspective for PD diagnosis if we want to test and possibly implement disease modifying therapies before motor symptoms become manifest.^40^

## Author Roles

(1) Research project: A. Conception, B. Organization, C. Execution; (2) Statistical Analysis: A. Design, B. Execution, C. Review and Critique; (3) Manuscript Preparation: A. Writing of the first draft, B. Review and Critique.

A.A.: 1 A C; 2 C; 3 B.

M.C.: 1 A C; 2 C; 3 A, B.

A.E.: 1 C; 2 C; 3 B.

## Disclosures

**Ethical Compliance Statement:** Informed patient consent was not necessary for this work, and the approval of an institutional review board was not required. We confirm that we have read the Journal's position on issues involved in ethical publication and affirm that this work is consistent with those guidelines.

**Funding Sources and Conflicts of Interest:** No specific funding was received for this work, and there are no conflicts of interest to declare.

**Financial Disclosure for Previous 12 Months:** A.A. has received compensation for consultancy and speaker‐related activities from UCB, Britannia, AbbVie, Zambon, Bial, Ever Pharma, Theravance Biopharma, Roche, General Electric, Medscape. He receives research support from Chiesi Pharmaceuticals, Lundbeck, Bial, Movement Disorders Society, Horizon2020 Grant 825785, Horizon2020 Grant 101016902, Ministry of Education University and Research (MIUR) Grant ARS01_01081, Cariparo Foundation. M.C. has received travel grants from Lusofarmaco and Zambon. A.E. nothing to declare.
